# Corrections to *Agrilus* related species–group names in the world catalogue of Bellamy and new substitute names for *Agrilus* species–group homonyms

**DOI:** 10.3897/zookeys.249.4387

**Published:** 2012-12-10

**Authors:** Eduard Jendek

**Affiliations:** 1 Canadian Food Inspection Agency, K.W. Neatby Bldg., 960 Carling Avenue, Ottawa, Ontario, K1A 0C6, Canada

**Keywords:** Coleoptera, Buprestidae, *Agrilus*, *Aphanisticus*, *Australodraco*, new synonyms, new substitute names, lectotype designation

## Abstract

The work treats 52 species–group names related to genus *Agrilus* incorrectly cited in the world catalogue of Bellamy (2008). The name *dimorphus* Théry, 1941 from the genus *Aphanisticus* and *mulleri* Théry, 1925 from the genus *Australodraco* are also treated. Four primary or secondary homonyms are replaced by substitute names. Most of the proposed changes refer to the availability, validity, spelling and authorship of the names. The following new nomenclatural acts are proposed: Four new substitute names for homonyms: *gola* Jendek for filiformis Gory & Laporte, 1839 not Herbst, 1801; *lukesi* Obenberger, 1936 for *modicus* Kerremans, 1892 not Solier, 1833; *thomsoni* Jendek for *impressipennis* Thomson not Uhler, 1855; *walkerianus* Jendek (*Aphanisticus*) for *sulcicollis* Walker not Lacordaire, 1835. New synonyms: *turei* Curletti, 2002 is an objective synonym of *thurei* Curletti, 1996. Lectotype designations: A lectotype is designated for *Agrilus dualaecola* Obenberger, 1923.

## Introduction

In 2008–2009, a monumental catalogue on world buprestid beetles was published by Chuck L. Bellamy. The major part of the Volume 4 (Bellamy 2008) is devoted to the genus *Agrilus*.

This work is aimed to clarify and correct some taxonomic and nomenclatural problems published in the catalogue. The term *Agrilus* related species–group names means names originally or subsequently assigned to the genus *Agrilus*.

All data were checked from original publications rather than to rely on data published in the Zoological Records. The vast majority of treated taxa belongs to *Agrilus*, but some taxa of other genera are also influenced (see Abstract).

In the course of work, several *Agrilus* species–group names were found primary or se-condary homonyms. These names are replaced by substitute names in the separate section.

Treated names are cited alphabetically. Each name has the corrected status in parenthesis with its generic assignment if other than *Agrilus*. Pages where and how a taxon is listed in Bellamy (2008) is cited on the second line. The treatment of each case is stated in the last paragraph.

## Corrections to the Bellamy’s catalog

*Agrilus acutipennis* Horn (unavailable name)

Pages 2216, 2258 – cited as an available synonym of *olivaceoniger* Fisher, 1928 on page 2216 and *quadriimpressus* on page 2258.

Remark. Horn (1891) did not propose the name as new, he cited *acutipennis* Mannerheim, 1837. The name *acutipennis* Horn is an unavailable name introduced by Fisher (1928).

*Agrilus aenescens* Kerremans, 1903 (valid name)

Page 1953 – cited as a synonym of *aenescentellus* Obenberger, 1936c.

Remark. The name *aenescentellus* Obenberger, 1936c was proposed as a replacement name for *aenescens* Kerremans, 1903 not Shilsky, 1888. Because the name *aenescens* Shilsky is unavailable (see Jendek 2002b), the name *aenescentellus* Obenberger is an unnecessary replacement name and junior objective synonym of *aenescens* Kerremans.

*Agrilus agadiensis* Théry, 1910 (valid name)

Page 2050 – cited as a synonym of *cupriventris* Gory & Laporte, 1839

Remark. The name *agadiensis* Théry, 1910 was cited by Curletti (1993) as a valid name of species. This latest taxonomic act has been omitted.

*Agrilus atriplicis* Escalera (unavailable synonym of *rabaticus* Théry, 1930)

Page 2261 – cited as available synonym of *rabaticus*

Remark. Escalera (1914) did not propose the name as new, he cited Abeille as the author. The name *atriplicis* Escalera is an unavailable name introduced in the synonymy of *Agrilus rabaticus* by Obenberger, 1936a.

*Agrilus bandanus* Obenberger, 1933 (unavailable synonym of *sulphurifer* Burmeister, 1872)

Page 2318 – cited as available synonym of *sulphurifer*

Remark. The name *bandanus* Obenberger, 1933 was proposed for an aberration, which is an unavailable name (ICZN, Article 45.6.2).

*Agrilus bayeri* Obenberger, 1935d (synonym of *sinensis splendidicollis* Fairmaire, 1889)

Page 2294 – cited as *beyeri*; synonym of *sinensis splendidicollis*

Remark. Original spelling of this patronymic name is *bayeri* (dedicated to Prof. E. Bayer) not *beyeri* which is incorrect subsequent spelling.

*Agrilus bolamanus* Kerremans (unavailable synonym of *buraicus* Obenberger, 1928)

Page 2010 – cited as available synonym of *buraicus*

Remark. Kerremans (1914) did not propose the name as new, he cited *Agrilus bolamanus* Kerremans 1906. The name *bolamanus* Kerremans, 1914 not Kerremans, 1906 is an unavailable name introduced in the synonymy of *Agrilus buraicus* by Obenberger (1928).

*Agrilus borrei* Kerremans (unavailable synonym of *deborrei* Dugès, 1891)

Page 2059 – cited as available synonym of *deborrei*

Remark. Kerremans (1892a) did not propose the name as new, he misspelled *Agrilus deborrei* Dugès, 1891. The name *borrei* Kerremans is an unavailable name introduced in the synonymy of *Agrilus deborrei* by Waterhouse (1897).

*Agrilus caliginulus* Obenberger, 1935e (valid name)

Page 2012 – cited as *calliginulus*

Remark. Original spelling of the name is *caliginulus* not *calliginulus* which is incorrect subsequent spelling.

*Agrilus cameroni* Bellamy, 1999 (unavailable name)

Page 2012 – cited as valid name for species

Remark. The name *cameroni* Bellamy, 1999 was proposed as a new replacement name for *dimorphus* Théry, 1941, which was supposed to be preoccupied by *dimorphus* Obenberger, 1923. Théry (1941), however, did not propose *dimorphus* in *Agrilus* but in *Aphanisticus* so the replacement was unnecessary. The names *Agrilus dimorphus* Théry and *Agrilus cameroni* Bellamy are unavailable names. See also notes at *dimorphus* Théry, 1941.

*Agrilus camerounensis* Obenberger (unavailable name)

Page 2012 – cited as *cameroonensis* and valid name for species

Remark. The name *camerounensis* Obenberger, 1931 is listed as a valid name of species but I failed to find original publication neither by Obenberger or anybody else. The name *camerounensis* Obenberger is an unavailable name introduced by Curletti (1993) .

*Agrilus capicola* Kerremans, 1898 (valid name)

Page 2013 – cited as *capicolus*

Remark. Original spelling of the name is *capicola* not *capicolus*. The name *is to be treated as a noun in apposition to the name of its genus* as ruled be ICZN (Article 31.2.2). The name *capicolus* is an incorrect subsequent spelling.

*coeruleoniger* Fisher, 1929 (valid name) Page 2032 – cited as *coeruleonigra*

Remark. Original spelling of the name is *coeruleonigra*. The ending has to be changed to *coeruleoniger* to agree with the genus gender. This is a mandatory change ruled by ICZN (Article 34.2).

*Agrilus confusulan* Obenberger, 1935a (unavailable synonym of *grandis* Gory & Laporte, 1839)

Page 2115 – cited as *confusulus* and unavailable synonym of *grandis*

Remark. Original spelling of this unavailable name proposed for an aberration is *confusulan*.

*Agrilus coronifrons* Obenberger, 1931a (valid name)

Page 2227 – cited as a synonym of *patricius*

Remark. I failed to trace any author who proposed the synonymisation of the names *patricius* Obenberger, 1931a and *coronifrons* Obenberger, 1931a. The last use of both names was that of Curletti (1998b) who cited them as valid names for species.

*Agrilus cyaneus* Olivier (unavailable synonym of *viridis* Linné, 1758) Page 2354 – cited as available synonym of *viridis*

Remark. Olivier (1790a) did not propose the name as new, he cited *Buprestis cyanea* Fabricius, 1775. The name *cyaneus* Olivier is an unavailable name introduced in the synonymy of *Agrilus viridis* by Schönherr (1817).

*Agrilus dicalis* Kerremans (unavailable synonym of *dualis* Kerremans, 1903)

Page 2069 – cited as valid name for species.

Remark. Kerremans never proposed the name *dicalis*. This name was introduced by Obenberger (1936) who misspelled the name *dualis* Kerremans, 1903. This lapse was repeated by Blackwelder (1944) and later by Bellamy (2008). The name *dicalis* Kerremans is an unavailable synonym of *dualis* Kerremans, 1903.

*Agrilus dimorphus* Théry, 1941 (*Aphanisticus*; valid name for species)

Page 2012, 2395 – cited as a synonym of *cameroni* Bellamy, 1999 in *Agrilus* on page 2012 and as a valid name for species in *Aphanisticus* on page 2395.

Remark. The name *dimorphus* Théry, 1941 is a valid name for species in *Aphanisticus*. See also remarks at the name *cameroni* Bellamy, 1989.

*Agrilus dualaecola* Obenberger, 1923 (synonym of *roscidinus* Obenberger, 1923)

Page 2268, 2310 – cited as a synonym of *roscidinus* on page 2268 and synonym of *subcurtulus* on page 2310.

Remark. Curletti (1993) examined 3 syntypes of *Agrilus roscidinus* subspecies *dualaecola* Obenberger, 1923 in National Museum , Prague, Czech Republic (NMPC) and found out that they belong to two different taxa. He treated the name *dualaecola* as a junior subjective synonym of *aterrimus* Kerremans, 1909 based on two female syntypes as well as a synonym of *roscidinus* Obenberger, 1923 based on a single male syntype. Bellamy followed this approach and cited the *dualaecola* also as a synonym of two different names (see above). In order to preserve the stability of nomenclature by fixing the status of the specimen as the sole name–bearing type of a particular nominal taxon, I designate herein the male syntype of *Agrilus roscidinus* subspecies *dualaecola* Obenberger, 1923 preserved in NMPC as a lectotype. The name *dualaecola* Obenberger, 1923 is a junior subjective synonym of *roscidinus* Obenberger, 1923.

*Agrilus cyanophilus* Schaefer, 1946 (unavailable synonym of *suvorovi* Obenberger, 1935)

Page 2320–2321, 2358 – cited as unavailable synonym of *suvorovi* on Page 2320 and as *cyanophila* an unavailable synonym of *viridis* on Page 2358

Remark. The name *cyanophila* Schaefer, 1946 was proposed for an aberration. The name with the correct ending *cyanophilus* is an unavailable synonym of *suvorovi* Obenberger, 1935 (see also Jendek 2002a).

*Agrilus enriquei* Murria Beltrán & Murria Beltrán, 2007 (valid name) Page 2083 – cited as *enriguei*

Remark. Original spelling of this patronymic name is *enriquei* (dedicated to Enrique Murria Beltrán) not *enriguei* which is incorrect subsequent spelling.

*Agrilus escalerai* Obenberger, 1921 (valid name) Page 2084 – cited as *escaleri*

Remark. Original spelling of this patronymic name is *escalerai* (dedicated to Escalera) not *escaleri* which is incorrect subsequent spelling.

*Agrilus ferrugineoguttatus* Gory & Laporte (unavailable synonym of *discolor* Fåhraeus, 1851) Page 2072 – cited as available synonym of *discolor*

Remark. Gory and Laporte (1839) did not propose the name as new, they cited *Agrilus ferrugineoguttatus* Herbst, 1801. The name *ferrugineoguttatus* Gory & Laporte is an unavailable name introduced in the synonymy of *Agrilus discolor* by Fåhraeus (1851).

*Agrilus fulgidicollellus* Obenberger, 1935 (unavailable name)

Page 2328 – cited as available synonym of *thoracicus*

Remark. Obenberger (1935c) proposed a replacement name *fulgidicollellus* for non–existing name *fulgidicollis* Fisher not Dejean. The name *fulgidicollellus* Obenberger is an unavailable name with no relation to the name *thoracicus* Gory & Laporte, 1839, as erroneously stated by Moore Rodriguez (1985).

*Agrilus goryi* Saunders, 1871 (valid name)

Page 2118 – cited as valid name proposed as a replacement name

Remark. The name *goryi* Saunders, 1871 is a name proposed by indication to the bibliographic reference of Laporte and Gory (1839) (see ICZN Article 12.2) and not a proposal of a new replacement name.

*guerryi* Obenberger, 1933 (valid name)

Page 2118 – cited as *guercyi*

Remark. Original spelling of this patronymic name is *guerryi* (dedicated to Guerry) not *guerrcyi* which is an incorrect subsequent spelling.

*Agrilus ingnoratus* Obenberger, 1924 (unavailable synonym of *ribbei* Kiesenwetter, 1879) Page 2266 – cited as available synonym of *ribbei*

Remark. Obenberger (1924) described an *Agrilus* which name was spelled as *ingnoratus* on page 44 and *ignoratus* on pages 45 and 46. Precedence of the name *ignoratus* was fixed by Obenberger (1926) as the first reviser (ICZN Article 24.2.4). The name *ingnoratus* is an incorect original spelling and unavailable name (ICZN Article 32.4)].

*Agrilus insulicola* Kerremans, 1912 (valid name)

Page 2141 – cited as *insulicolus*

Remark. Original spelling of this name is *insulicola* not *insulicolus*. The name *is to be treated as a noun in apposition to the name of its genus* as ruled be ICZN (Article 31.2.2). The name *insulicolus* is an incorrect subsequent spelling.

*Agrilus javicola* Fisher, 1935 (valid name)

Page 2146 – cited as *javicolus*

Remark. Original spelling of this name is *javicola* not *javicolus*. The name *is to be treated as a noun in apposition to the name of its genus* as ruled be ICZN (Article 31.2.2). The name *javicolus* is an incorrect subsequent spelling.

*Agrilus kachovskii* Obenberger, 1935e (valid name)

Page 2148 – cited as *kackovskii*

Remark. Original spelling of this patronymic name is *kachovskii* (dedicated to Kachovski) not *kackovskii* which is incorrect subsequent spelling.

*Agrilus linearis* Panzer (unavailable synonym of *viridis* Linné, 1758)

Page 2354 – cited as available synonym of *viridis*

Remark. Panzer (1806) did not propose the name as new, he cited *Buprestis linearis* Fabricius, 1792. The name *linearis* Panzer is an unavailable name introduced in the synonymy of *Agrilus viridis* by Kiesenwetter (1857).

*Agrilus mucronatus* Gory & Laporte (unavailable synonym of *goryi* Saunders, 1871)

Page 2110 – cited as available synonym of *goryi*

Remark. Gory and Laporte (1839) did not propose the name as new, they cited *Agrilus mucronatus* Klug, 1825. The name *mucronatus* Gory & Laporte is an unavailable name introduced in the synonymy of *Agrilus goryi* by Saunders (1871).

*Agrilus muscarinus* Baudon (unavailable synonym of *muscarius* Kerremans, 1895)

Page 2196 – cited as available synonym of *muscarius*

Remark. Baudon (1960) did not propose the name as new, he misspelled *Agrilus muscarius* Kerremans, 1895. The name *muscarinus* Baudon is an unavailable name introduced in the synonymy of *Agrilus muscarius* by Descarpentries and Villiers (1963).

*Agrilus mulleri* Théry, 1925 (*Australodraco*; valid name)

Page 2369 – cited as *muelleri*

Remark. Original spelling of this patronymic name is *mulleri* (dedicated to Franklin Müller) not *mülleri* which would authorized the mandatory change of *ü* to *ue* (ICZN, Article 32.5.2). The name *muelleri* is an incorrect subsequent spelling.

*Agrilus novicus* Westhoff (unavailable synonym of *viridis* Linné, 1758)

Page 2356 – cited as available synonym of *viridis*

Remark. Westhoff (1881) did not propose the name as new, he misspelled the name *nocivus* Ratzeburg, 1837. The name *novicus* Westhoff is an unavailable name introduced in the synonymy of *Agrilus viridis* by Obenberger (1936a).

*Agrilus otini* Théry, 1934 (synonym of *lukuledianus* Kerremans, 1907)

Page 2172 – cited as subspecies of *lukuledianus*

Remark. The act of Curletti (1993) who cited *otini* Théry, 1934 as a junior synonym of *lukuledianus* Kerremans, 1907 was overlooked.

*Agrilus parapupala* Curletti, 1998a (valid name)

Page 2225 – cited as *parapupalus*

Remark. Curletti (1998a) proposed the new species name *Agrilus parapupala* in order to stress its similarity with *Agrilus pupala* Obenberger, 1935e. The name *parapupalus* is an incorrect subsequent spelling. See also *pupala*.

*Agrilus patrizii* Théry, 1927 (valid name)

Page 2227 – cited as *patrizzii*

Remark. Original spelling of this name is *patrizii* not *patrizzii* which is an incorrect subsequent spelling.

*Agrilus politus* Weiss (unavailable synonym of *cuprescens* Ménétriés, 1832)

Pages 2048, 2356 – cited as unavailable synonym of *cuprescens* on page 2048 and as unavailable synonym of *viridis* on page 2356

The unavailable name *politus* cited by Weiss (1914) is a misidentification of *Agrilus cuprescens* not *Agrilus viridis* (Linné, 1758).

*Agrilus pseudroberti* Fleischer, 1934 (unavailable synonym of *cyanescens* Ratzeburg, 1837)

Page 2055 – cited as *pseudoroberti*; unavailable synonym of *cyanescens*

Remark. The original spelling of this unavailable name proposed by Fleischer (1934) for an aberration is *pseudroberti* not *pseudoroberti*.

*pupala* Obenberger, 1935e (valid name)

Page 2255 – cited as *pupalus*

Remark. Original spelling of the name is *pupala* not *pupalus*. Following the ICZN, Article 31.2.2., the name is to be treated as a noun in apposition and original spelling is to be retained. The *pupalus* is incorrect subsequent spelling. See also *parapupala*.

*Agrilus pygaera* Obenberger (unavailable name)

Page 2257 – cited as valid name of species

Remark. Obenberger (1931b) did not propose *pygaera* in the genus *Agrilus* but in *Anthaxia*. Ferreira (1963) erroneously cites the name in *Agrilus*. Bellamy (2008) followed the lapse of Ferreira. The name *Agrilus pygaera* Obenberger is an unavailable name.

*Agrilus pyri* Blanchard, 1845 (available synonym of *sinuatus* Olivier, 1790b)

Page 2296 – cited as unavailable synonym of *sinuatus*

Remark. Blanchard (1845) cited name *pyri* without characters but the species is illustrated on the Plate 9, Fig 15. According the ICZN Article 12.2.7, the name published before 1931 without characters but associated with an illustration of the taxon being named is an available name.

*Agrilus rugiplumbeus* Cobos, 1964 (valid name)

Page 2274 – cited as *rugiplumbus*

Remark. Original spelling of this name is *rugiplumbeus* not *rugiplumbus* that is incorrect subsequent spelling.

*Agrilus shamyl* Obenberger, 1922 (subspecies of *lineola* Kiesenwetter, 1857)

Page 2167 – cited as *shamyi*; subspecies of *lineola*

Remark. Original spelling of the name is *shamyl* not *shamyi* which is an incorrect subsequent spelling.

*Agrilus sibiricola* Obenberger, 1924 (synonym of *laticornis* Illiger, 1803)

Page 2291 – cited as *sibiricolus*

Remark. Original spelling of this name is *sibiricola* not *sibiricolus*. The name *is to be treated as a noun in apposition to the name of its genus* as ruled be ICZN (Article 31.2.2). The name *sibiricolus* is an incorrect subsequent spelling.

*Agrilus spiniger* Gory & Laporte, 1839 (available synonym of *spinamajor* Chevrolat, 1838) Page 2302 – cited as unavailable synonym of *spinamajor*

Remark. Gory and Laporte (1839) proposed *spiniger* as a name for new species. The name is junior secondary homonym to *spiniger* Eschscholtz, 1822. Lacordaire (1857) put it to the synonymy of *Agrilus spinamajor* Chevrolat, 1838.

*Agrilus sulcifer* Bétis (unavailable synonym of *hyperici* Creutzer, 1799)

Page 2130 – cited as available synonym of *hyperici*

Remark. Bétis (1908) did not propose the name as new, he cited *Agrilus sulcifer* Abeille de Perrin, 1895. The name *sulcifer* Bétis is an unavailable name introduced in the synonymy of *Agrilus hyperici* by Schaefer (1949).

*Agrilus thurei* Curletti, 1996 (valid name) correct original spelling

Page 2337 – cited as a synonym of *turei*

Remark. The name *thurei* Curletti, 1996 was originally dedicated to A. Thure which follows from the *derivatio nominis* as well as from the name of collector cited on the labels of type specimens. The name was changed by Curletti (2002) to *turei* based on the fact that the verified name orthography is *Ture* not *Thure*.

Following the ICZN (Article 32.4): the original spelling of a name is the “correct original spelling”, unless it is demonstrably incorrect as provided in Article 32.5. The Article 32.5 *requires clear evidence of an inadvertent error in the original publication itself without recourse to any external source of information*. The publication of Curletti (1996) itself doesn’t provide such an evidence of an inadvertent error. The case is further intricate by the fact that Bellamy (2008) cited the original spelling of the name (*thurei*) as a synonym of the emended name (*turei*). If the nomenclatural act of Curletti (1996) had been a justified emendation then the emended name (*turei*) would have to take the authorship and date of the original publication (ICZN Article 19.2); and the incorrect original spelling sensu Curletti (*thurei*) would have no separate availability (Article 32.4).

The best solution for the stability of the nomenclature is the reversal to original state by following the ICZN Article 32.5. The name *thurei* Curletti, 1996 is a correct original spelling and the name *turei* Curletti, 2002 an unjustified emendation and available synonym of *thurei*.

*Agrilus turei* Curletti, 2002 (available synonym of *thurei* Curletti, 1996) unjustified emendation

Page 2337 – cited as valid name

Remark. See comments at *thurei* above.

*Agrilus viridis* Seidlitz (unavailable synonym of *cuprescens* Ménétriés, 1832)

Pages 2047, 2356 – cited as unavailable synonym of *cuprescens* on page 2047 and unavailable synonym of *viridis* on page 2356

Remark. Seidlitz (1888) did not propose the name as new, he cited *Agrilus viridis* Linné, 1758. The name *viridis* Seidlitz is an unavailable name introduced by Obenberger (1935b) as a synonym of *rubicola* Abeille de Perrin, 1897, which is currently a synonym of *cuprescens* Ménétriés, 1832.

*Agrilus zanthoxylumi* Li Meng Lou, 1989 (valid name)

Page 2367 – Zhang and Wang are cited as authors.

Remark. The authorship of the name has been changed several times. Jendek & Grebennikov (2011) stated that Li Meng Lou (1989) is the first who established the name by presenting characters (ICZN, Article 13.1.1).

## Substitute names for primary homonyms in the genus *Agrilus*

*Agrilus lukesi* Obenberger, 1936b new substitute name

Remark. The name *modicus* Kerremans, 1892b is replaced by its synonym *lukesi* Obenberger, 1936b due to primary homonymy with *modicus* Solier, 1833 (recently in *Paragrilus*).

*Agrilus thomsoni* Jendek new replacement name

Remark. The name is *impressipennis* Thomson, 1879 is replaced by a new replacement name *thomsoni* Jendek due to primary homonymy with *impressipennis* Uhler, 1855 (currently synonym of *fallax* Say, 1833).

Etymology: The name is patronymic and dedicated to Thomson.

*Agrilus walkerianus* Jendek (*Aphanisticus*) new replacement name

Remark. The name *sulcicollis* Walker, 1858 (recently in *Aphanisticus*) is replaced by a new replacement name *walkerianus* Jendek due to primary homonymy with *sulcicollis* Lacordaire, 1835.

Etymology: The name is patronymic and dedicated to Walker.

## Substitute name for secondary homonyms in the genus *Agrilus*

*Agrilus gola* Jendek new replacement name

Remark. The name *filiformis* Gory & Laporte, 1839 (originally proposed in *Agrilus*) is replaced by a new replacement name *gola* Jendek due to primary homonymy with *filiformis* Herbst, 1801 (originally proposed in *Buprestis*; currently synonym of *viridis* Linné, 1758).

Etymology: The name is derived from the first two letters of the names Gory and Laporte.

## References

[B1] Abeille de Perrin E (1895) Buprestides paléarctiques réputés nouveaux. Annales de la Société Entomologique de France 64: 116-126.

[B2] Abeille de Perrin E (1897) Notes sur les Buprestides paléarctiques (Suite). Revue d’Entomologie 16: 1-33.

[B3] Baudon A (1960) Contribution à l’étude des Buprestides du Laos. Annales de la Faculté des Sciences de Saigon 1960: 217-233.

[B4] Bellamy CL (1999) Nomenclatural notes in Buprestidae (Coleoptera). Folia Heyrovskyana 7 (1): 1-11.

[B5] Bellamy CL (2008) A world catalogue and bibliography of the jewel beetles (Coleoptera: Buprestoidea). Volume 4, Agrilinae: Agrilina through Trachyini. Pensoft, Sofia, 1932–2684.

[B6] Bétis L (1908) Synopsis des Coléoptères du Var. Draguignan, Imprimerie Latil Freres, 971 pp.

[B7] Blackwelder RE (1944) Checklist of the coleopterous insects of Mexico, Central America, The West Indies, and South America. United States National Museum, Bulletin 185 (2): 189-341.

[B8] Blanchard F (1845) Histoire naturelle des insectes : leurs moeurs, leurs métamorphoses, et leur classification ou traite elementaire d’entomologie. Tome Second, F. Savy, Paris, 524 pp.

[B9] Burmeister HCC (1872) Buprestidae Argentini, Uebersicht der Prachtkäfer des La Plata-Gebietes. Stettiner Entomologische Zeitung 33: 367-387.

[B10] Chevrolat LAA (1838) Centurie de Buprestides. Silbermann’s Revue Entomologique, Strasbourg and Paris, 5: 41-110.

[B11] Cobos A (1964) Nuevas especies de Buprestidae (Coleoptera) de Nueva Guinea. Annali del Museo Civico di Storia Naturale di Genova 75: 197-212.

[B12] Creutzer C (1799) Entomologische Versuche. Karl Schaumburg und Comp., Wien, 142 pp.

[B13] Curletti G (1993) First contribution to the revision of the genus *Agrilus* of the Ethiopic region (Coleoptera, Buprestidae). Introduction – Checklist – First synonymies – New Subgenera. Lambillionea 93: 421-444.

[B14] Curletti G (1996) Nuovi taxa Africani del gen. *Agrilus* Curtis, 1825. VI. (Coleoptera: Buprestidae). Elytron, Barcelona 10: 123-134.

[B15] Curletti G (1998a) Nuovi *Agrilus* delle regioni Africane e delle penisola Arabica (Coleoptera, Buprestidae). Rivista Piemontese di Storia Naturale 19: 89-149.

[B16] Curletti G (1998b) Notes on metatarsal morphology in the genus *Agrilus* and proposed redefinition of its subgenera in afrotropical region (Coleoptera Buprestidae). Bolletino della Società entomologica italiana 130 (2): 125-134.

[B17] Curletti G (2002) Contribution a la connaissance des Agrilini de Cote d’Ivoire (Coleoptera: Buprestidae). Lambillionea 102: 43-64.

[B18] Descarpentries A Villiers A (1963) Catalogue raisonné des Buprestidae d’Indochine. II. Agrilini, genre *Agrilus* (2e partie). Revue française d’Entomologie 30 (2): 104-119.

[B19] Dugès DE (1891) Descripcion de coleopteros indigenas. La Naturaleza Serie 2, 2: 1–38.

[B20] Escalera MM de la (1914) Los Coleópteros de Marruecos. Trabajos del Museo Nacional de Ciencias Naturales, Madrid Serie Zoológica 11: 1-553.

[B21] Eschscholtz JF (1822) Entomographien. Erste Lieferung. G. Reimer, Berlin, 128 pp.

[B22] Fabricius JC (1775) Systema entomologiae, sistens insectorum classes, ordines, genera, species, adiectis synonymis, locis, descriptionibus, observationibus. Flensburgi et Lipsiae, Libraria Kortii, 832 pp.

[B23] Fabricius JC (1792) Entomologia systematica emendata et aucta, secundum classes, ordines, genera, species adjectis synonimis, locis, observationibus, descriptionibus. Tom. I. Pars II, Hafniae, Christ. Gottl. Proft. 538 pp. doi: 10.5962/bhl.title.36532

[B24] Fairmaire L (1889) Descriptions de Coléoptères de l’Indo-Chine. Annales de la Société Entomologique de France 6 (8): 333-378.

[B25] Fåhraeus OI (1851) [New buprestid taxa]. In: Boheman CH (Ed.). Insecta Caffrariae annis 1838–1845 a J. H. Wahlberg collecta, amici auxilio suffultus. Pars I. Fascic. II. Coleoptera. (Buprestides, Elaterides, Cebrionites, Rhipicerides, Cyphonides, Lycides, Lampyrides, Telephorides, Malyrides, Clerii, Terediles, Ptiniores, Palpatores, Silphales, Histeres, Scaphidilia, Nitidulariae, Cryptophagidae, Byrrhii, Dermestini, Parnidae, Hydrophylidae) Holmiae, Norstedtiana: 299-374.

[B26] Ferreira MC (1963) Catálogo dos Coleópteros de Moçambique. Revista de Entomologia de Moçambique 6 (1): 1-532.

[B27] Fisher WS (1928) A revision of the North American species of the buprestid beetles belonging to the genus *Agrilus*. Bulletin of the United States National Museum 145: 1-347. doi: 10.5479/si.03629236.145.1

[B28] Fisher WS (1929) New species of buprestid beetles from Costa Rica. Proceedings of the United States National Museum No. 2803, 76(6): 1–20.

[B29] Fisher WS (1935) New species of Buprestidae from Java (Col.). Treubia 15 (1): 27-48.

[B30] Fleischer A (1934) Přehled brouků fauny Československé republiky. Zvláštní otisk z Časopisu Moravského Musea Zemského, ročník 25-27. Akciová Moravská knihtiskárna, Brno, 483 pp.[in Czech]

[B31] Gory HL Laporte de Castelnau FL (1839) Histoire naturelle et iconographie des insectes coléoptères, publiée par monographies séparées. Suite aux buprestides. Tome II, P. Duménil, Paris. [genera paged separately]

[B32] Herbst JFW (1801) Natursystem aller bekannten in– und ausländischen Insekten, als eine Fortsetzung der Büffonschen Naturgeschichte. Der Käfer. Neunter Theil. Buchhandlung des Geh. Commerzien–Raths Bauti, Berlin, 344 pp.

[B33] Horn GH (1891) The species of *Agrilus* of Boreal America. Transactions of the American Entomological Society 18: 277-366.

[B34] ICZN (1999) International Code of Zoological Nomenclature. Fourth Edition, adopted by the International Union of Biological Sciences. International Trust for Zoological Nomenclature, London, 306 pp.

[B35] Illiger JCW (1803) Verzeichniss der in Portugall einheimischen Käfer. Erste Lieferung. Magazin für Insektenkunde, Braunschweig 2: 186-258.

[B36] Jendek E (2002a) Taxonomic and nomenclatural notes on *Agrilus suvorovi* Obenberger (Coleoptera: Buprestidae: Agrilinae). Zootaxa 52: 1-8.

[B37] Jendek E (2002b) Nomenclatural and taxonomic notes on *Agrilus ater* (Linné), *A. biguttatus* (Fabricius) and *A. subauratus* Gebler (Coleoptera: Buprestidae: Agrilinae). Zootaxa 120: 1-12.

[B38] Jendek E Grebennikov V (2011)*Agrilus* (Coleoptera, Buprestidae) of East Asia. Prague, Jan Farkač, 362 pp.

[B39] Kerremans C (1892a) Catalogue synonymique des Buprestides decrits de 1758 à 1890. Séance du 5 septembre 1891. Mémoires de la Société Entomologique de Belgique 1: 1-304.

[B40] Kerremans C (1892b) Viaggio di Leonardo Fea in Birmania e regioni vicine. XLIX. Buprestides. Annali del Museo Civico di Storia Naturale di Genova Serie 2, 12(32): 809–832.

[B41] Kerremans C (1895) Buprestides Indo–Malais. Deuxième partie. Annales de la Société Entomologique de Belgique 39: 192-224.

[B42] Kerremans C (1898) Buprestides du Congo et des régions voisines. Annales de la Société Entomologique de Belgique 42: 271-329.

[B43] Kerremans C (1903) Coleoptera Serricornia. Fam. Buprestidae, Fasc. 12b, 12c, 12d. In: Wytsman P (Ed.). Genera Insectorum. Tome II, Fascicules XII–XIV. Verteneuil & Desmet, Bruxelles: 49-338.

[B44] Kerremans C (1906) Buprestides recueillis par Mr. L. Fea dans l’Afrique occidentale. Annali del Museo Civico di Storia Naturale di Genova Serie 3, 2(42): 406–411.

[B45] Kerremans C (1907) Buprestides de l’Est Africain allemand. Annales de la Société Entomologique de Belgique 51: 60-67.

[B46] Kerremans C (1909) Catalogues raisonnés de la Faune Entomologique du Congo Belge. Coleoptera, fam. Buprestidae. Annales du Musée du Congo Belge Zool. ser. III, tome I, fasc. 2, 1–44.

[B47] Kerremans C (1912) H. Sauter’s Formosa–Ausbeute. Buprestiden. Archiv für Naturgeschichte 78, Abteilung A, Heft 7: 203–209.

[B48] Kerremans C (1914) Buprestidae. In: Voyage de Ch. Alluad et R. Jeannel en Afrique orientale (1911–1912). Résultats scientifiques, Memoire No. 28, Coleoptera VI, Libraire A. Schulz, Paris, 207–246.

[B49] Kiesenwetter H von (1857) Buprestidae, Eucnemidae, Bogen 1–11, Naturgeschichte der Insecten Deutschlands begonnen von Dr. W. F. Erichson, fortgesetzt von Prof. Dr. H. Schaum, Dr. G. Kraatz und H. v. Kiesenwetter. Erste Abtheilung Coleoptera. Vierter Band. Berlin, Nicolaische Verlagsbuchhandlung (G. Parthey), 1–178.

[B50] Klug JCF (1825) Entomologiae Brasilianae specimen alterum, sistens insectorum coleopterorum nondum descriptorum centuriam. Nova Acta Physico–Medica Academiae Caesareae Leopoldino–Carolinae, Naturae curiosum, Bonnae Tomus duodecimus seu decadis secundae tomus secundus, 419–476.

[B51] Lacordaire MT (1835) [The volume on Beetles]. In: Boisduval JBA Lacordaire MT (Eds). Faune entomologique des environs de Paris, ou species général des insectes qui se trouvent dans un rayon de quinze a vingt lieues aux alentours de Paris. Tome premier. Méquignon–Marvis, Paris: 103-696.

[B52] Lacordaire MT (1857) Histoire naturelle des insectes. Genera des coléoptères ou exposé méthodique de critique de tous les genres proposés jusqu’ici dans cet ordre d’insectes. Tome quatrième contenant les familles des buprestides, throscides, eucnémides, élatérides, cébrionides, cérophytides, rhipicérides, dascyllides, malacodermes, clérides, lyméxylones, cupésides, ptiniores, bostrichides et cissides. Libraire encyclopédique de Roret, Paris, 554 pp.

[B53] Li Meng Lou (1989) *Agrilus zanthoxylumi*. In: Lou LM Min CZ Xin WP (Eds). Growth and pest control of Zanthoxylum bungeanum Maxim. Xi’an, Shaanxi Science & Technology Press: 60-63.

[B54] Linné C von (1758) Systema naturae per regna tria naturae, secundum classes, ordines, genera, species, cum characteribus, differentiis, synonymis, locis. Editio decima, reformata. Holmiae, Impensis Direct. Laurentii Salvii, 824 pp.doi: 10.5962/bhl.title.542

[B55] Mannerheim CG von (1837) Enumeration des Buprestides, et description de quelques nouvelles espèces de cette tribu de la famille des Sternoxes, de la collection de M. Le Comte Mannerheim. Bulletin de la Société Impériale des Naturalistes de Moscou 10: 1-126.

[B56] Ménétriés E (1832) Catalogue raissonné des objets de Zoologie recueillis dans un voyage au Caucase et jusqu’aux frontières actuelles de la Perse. L’Imprimerie de l’Académie Impériale des Sciences, St. Pétersbourg, 271 pp.

[B57] Moore Rodriguez T (1985) Aporte al conocimiento de los Buprestidos de Chile (Coleoptera–Buprestidae) Segunda Nota. Revista Chilena de Entomología 12: 113-139.

[B58] Murria Beltrán F Murria Beltrán Á (2007) Descripción de una nueva especie del género *Agrilus* Curtis, 1825, de Espańa (Coleoptera, Buprestidae, Agrilinae). Boletin de la SEA, Sociedad Entomologica Aragonesa, Zaragoza No 40: 179-181.

[B59] Obenberger J (1921) Buprestides nouveaux de Fernando Poo et de la Guinée espagnole. Trabajos del Museo Nacional de Ciencias Naturales, Madrid Serie Zoológia 45: 1-16.

[B60] Obenberger J (1922) De novis Buprestidarum regionis palaearcticae speciebus II. Časopis Československé Společnosti Entomologické 19: 18–29, 66–71.

[B61] Obenberger J (1923) Eine Serie neuer Buprestidenarten. Tijdschrift voor Entomologie 66: 1-32.

[B62] Obenberger J. (1924) Symbolae ad specierum regionis palaearcticae Buprestidarum cognitionem. Jubilejní Sborník Československé Společnosti Entomologické 1924: 6-59.

[B63] Obenberger J (1926) Buprestidae, p. 620–663, In: Winkler A (Ed). Catalogus Coleopterorum regionis palaearcticae. Pars 6, A. Winkler, Wien: 625-752.

[B64] Obenberger J (1928) Sur les Buprestides recueillis par MM. Alluaud et Jeannel dans la region de Kilimandjaro (Col., Bupr.). Revise sbírky krasců, nalezených. Alluaudem a Jeannelem na Kilimandžaru. Acta Entomologica Musaei Nationalis Pragae 6: 3-28.

[B65] Obenberger J (1931a) Agrilenstudien I. (Col. Bupr.). Entomologisches Nachrichtenblatt 5 (3): 54-71.

[B66] Obenberger J (1931b) Studien über die aethiopischen Buprestiden II. Folia Zoologica et Hydrobiologica 3 (1): 84-127.

[B67] Obenberger J (1933) Několik poznámek o krascích. Quelquues notes et rectification sur les Buprestides (Col.). Časopis Československé Společnosti Entomologické 30: 13-17.

[B68] Obenberger J (1935a) De regionis aethiopicae speciebus generis Agrili novis (Col. Bupr.). Nové druhy krasců z rodu *Agrilus* z aethiopické oblasti (Col. Bupr.). Acta Entomologica Musaei Nationalis Pragae 13: 149-210.

[B69] Obenberger J (1935b) Catalogue raisonné des Buprestides de Bulgarie. III Partie. Bulletin des Institutions Royales d’Histoire Naturelle, Sofia 8: 23-96.

[B70] Obenberger J (1935c) Synonymia Agrilorum (Col. Bupr.) II. Časopis Československé Společnosti Entomologické 32: 121-121.

[B71] Obenberger J (1935d) De regionis palaearcticae generis Agrili speciebus novis (Col. Bupr.). O nových palaearktických druzích krasců z rodu *Agrilus*. Časopis Československé Společnosti Entomologické 32: 161-171.

[B72] Obenberger J (1935e) De generis Agrilis Africae speciebus novis (Col. Bupr.). Folia Zoologica et Hydrobiologica 8: 191-226.

[B73] Obenberger J (1936a) Buprestidae V. In: Junk W Schenkling S (Eds). Coleopterorum Catalogus, Volumen XIII, Pars 152, Verlag für Naturwissenschaften, W. Junk, Gravenhage: 935-1246.

[B74] Obenberger J (1936b) De speciebus novis palaearcticis generis *Agrilus* (Col. Bupr.). Nové palaearktické druhy rodu Agrilus (Col. Bupr.). Časopis Československé Společnosti Entomologické 33: 104-118.

[B75] Obenberger J (1936c) Synonymia Agrilorum (Col. Bupr.) II. Časopis Československé Společnosti Entomologické 33: 91-92.

[B76] Olivier AG (1790a) Encyclopedie méthodique. Histoire naturelle. Insectes. Tome cinquieme. Part 1, Paris, Panckoucke, 1–368.

[B77] Olivier AG (1790b) Entomologie, ou histoire naturelle des insectes, avec leurs caractères génériques et spécifiques, leur description, leur synonymie, et leur figure enluminée. Coléoptères. Tome second. Paris, De l’Imprimiere de Baudouin, Imprimeur de l’Assemblée Nationale, Genera No. 9–33. [paged separately]

[B78] Panzer GWF (1806) Fauna Insectorum Germaniae initia oder Deutschlands Insecten. Heft 101, Nürnberg, Felsecker, 24 pp.

[B79] Ratzeburg JTC (1837) Die Forst–Insecten oder Abbildung und Beschreibung der in den Wäldern Preussens und der Nachbarstaaten als schädlich oder nützlich bekannt gewordenen Insekten. In systematischer Folge und mit besonderer Rücksicht auf die Vertilgung der Schädlichen. Erster Theil. Die Käfer. Berlin, Nicolai Buchhandlung, Druckerei der Königlichen Akademie der Wissenschaften, 202 pp.

[B80] Saunders E (1871) Catalogus Buprestidarum Synonymicus et Systematicus. London, E. W. Janson, 171 pp.

[B81] Say T (1825) Descriptions of new American species of the genera *Buprestis*, *Trachys* and *Elater*. Annals of the Lyceum of New York 1, 249–268.

[B82] Say T (1833) Descriptions of new species of North American insects, and observations on some of the species already described. New Harmony, Indiana, 58–73.

[B83] Schaefer L (1946) Formes nouvelles de Buprestides. Bulletin mensuel de la Société Linnéenne de Lyon 6: 71-73.

[B84] Schaefer L (1949) Les Buprestides de France. Tableaux analytiques des Coléoptères de la faune franco–rhénane. France, Rhénane, Belgique, Hollande, Valais, Corse. Famille LVI. Miscellanea Entomologica Supplément, 511 pp, 28 pls.

[B85] Schilsky FJ (1888) Beitrag zur Kenntnis der deutschen Käferfauna. Deutsche Entomologische Zeitschrift 32: 177-190.

[B86] Schönherr CJ (1817) Synonymia Insectorum, oder Versuch einer Synonymie aller bisher bekannten Insecten; nach Fabricii Systema Eleutheratorum etc. geordnet. Eleutherata oder Käfer. Erster Band. Dritter Theil. Hispa – Molorchus. Upsala, Em. Bruzelius, 506 pp.

[B87] Seidlitz GCM (1888) Fauna Transsylvanica. Die Kaefer (Coleoptera) Siebenbürgens. 1.–2. Lieferung, Königsberg, Hartungsche Verlagsdruckerei, I–XL [introduction and families] + 1–48 [genera] + 1–240 [species] pp.

[B88] Solier AJJ (1833) Essai sur les Buprestides. Annales de la Société Entomologique de France 2: 261-316.

[B89] Théry A (1910) Voyage de M. Ch. Alluaud au Soudan Égyptien (Nil Bleu) (1905–1906). Buprestidae (Col.). Annales de la Société Entomologique de France 79 (3): 370-376.

[B90] Théry A (1925) Wissenschaftliche Ergebnisse der Bearbeitung der Coleopteren–Sammlung von Franklin Müller (Beitrag II). Buprestides. Entomologische Mitteilungen 14 (2): 162-165.

[B91] Théry A (1927) Buprestides de la Somalie Italienne récolts par le Marquis Patrizi. Annali del Museo Civico di Storia Naturale di Genova 52: 239-245.

[B92] Théry A (1930) Études sur les Buprestides de l’Afrique du nord. Mémoires de la Société des Sciences Naturelles du Maroc 19 (1928): 1-586.

[B93] Théry A (1934) Contributions a l’étude de la faune de Mozambique. Voyage de M. P. Lesne (1928–1929). 15e note, Coléoptères, Buprestides. Memórias e Estudos do Museu Zoológico de Universidade de Coimbra (1) 77: 1–31.

[B94] Théry A (1941) Notes on the Buprestidae (Coleoptera) of East Africa. Journal of the East Africa and Uganda Natural History Society 15: 91-153.

[B95] Thomson J (1879) Typi buprestidarum musaei Thomsoniani. Appendix 1a. Paris, E. Deyrolle, 87 pp.

[B96] Uhler PR (1855) Descriptions of a few species of Coleoptera supposed to be new. Proceedings of the Academy of Natural Sciences of Philadelphia 7: 415-418.

[B97] Walker F (1858) Characters of some apparently undescribed Ceylon Insects. Annals and Magazine of Natural History, Third series, Vol. 2: 280-286.

[B98] Waterhouse CO (1897) Biologia Centrali–Americana, Insecta, Coleoptera, Vol. III. Part 1, Serricornia, Buprestidae, Appendix. Fam. Buprestidae, 663–666.

[B99] Weiss HB (1914)*Agrilus politus* Say infesting roses. Journal of Economic Entomology 7: 251, 438–440.

[B100] Westhoff F (1881) Die Käfer Westfalens. I. Abtheilung. Bonn, Max Cohen & Sohn (Fr. Cohen). Verhandlungen des naturhistorischen Vereins der preussichen Rheinlande und Westfalens, Bonn Supplement, 38, Vierte Folge: 8. Jahrgang, XXVIII + 323 pp.

